# Retraction Note to: TREM-1 associated macrophage polarization plays a significant role in inducing insulin resistance in obese population

**DOI:** 10.1186/s12967-020-02365-1

**Published:** 2020-05-13

**Authors:** Saravanan Subramanian, Pradeep K. Pallati, Poonam Sharma, Devendra K. Agrawal, Kalyana C. Nandipati

**Affiliations:** 1 Department of Clinical and Translational Science, Creighton University School of Medicine, Omaha, NE USA; 2 Department of Surgery, Creighton University School of Medicine, 601 N. 30th Street, Suite # 3700, Omaha, NE 68131 USA; 3 Department of Pathology, Creighton University School of Medicine, Omaha, NE USA

## Retraction Note to: J Transl Med (2017) 15:85 10.1186/s12967-017-1187-7

This article [1] has been retracted at the request of the authors. Upon re-review of the data, we realized certain patients did not meet inclusion criteria. Per IRB guidelines, after removing three subjects who either had BMI > 65 or BMI < 40 (even though they had undergone bariatric surgery) and one control subject from the study, we re-analyzed the data set. The removal of these subjects from the analysis had no effect on our findings or conclusions. The journal’s Editor-in-Chief has invited the authors to re-publish their study with updated results.

All authors agree to this retraction.
